# VAMP4 is required to maintain the ribbon structure of the Golgi apparatus

**DOI:** 10.1007/s11010-013-1652-4

**Published:** 2013-05-16

**Authors:** Akiko Shitara, Toru Shibui, Miki Okayama, Toshiya Arakawa, Itaru Mizoguchi, Yasunori Shakakura, Taishin Takuma

**Affiliations:** 1 Division of Biochemistry, Department of Oral Biology, School of Dentistry, Health Sciences University of Hokkaido, Tobetsu Hokkaido, Ishikari, 061-0293 Japan; 2 Division of Anatomy, Department of Oral Growth and Development, School of Dentistry, Health Sciences University of Hokkaido, Tobetsu Hokkaido, Ishikari, 061-0293 Japan; 3 Division of Orthodontics and Dentofacial Orthopedics, Department of Oral Growth and Development, School of Dentistry, Health Sciences University of Hokkaido, Tobetsu Hokkaido, Ishikari, 061-0293 Japan

**Keywords:** VAMP4, SNARE, Golgi ribbon structure, Golgi fragmentation, RNAi, Membrane transport

## Abstract

The Golgi apparatus forms a twisted ribbon-like network in the juxtanuclear region of vertebrate cells. Vesicle-associated membrane protein 4 (VAMP4), a v-SNARE protein expressed exclusively in the vertebrate *trans*-Golgi network (TGN), plays a role in retrograde trafficking from the early endosome to the TGN, although its precise function within the Golgi apparatus remains unclear. To determine whether VAMP4 plays a functional role in maintaining the structure of the Golgi apparatus, we depleted VAMP4 gene expression using RNA interference technology. Depletion of VAMP4 from HeLa cells led to fragmentation of the Golgi ribbon. These fragments were not uniformly distributed throughout the cytoplasm, but remained in the juxtanuclear area. Electron microscopy and immunohistochemistry showed that in the absence of VAMP4, the length of the Golgi stack was shortened, but Golgi stacking was normal. Anterograde trafficking was not impaired in VAMP4-depleted cells, which contained intact microtubule arrays. Depletion of the cognate SNARE partners of VAMP4, syntaxin 6, syntaxin 16, and Vti1a also disrupted the Golgi ribbon structure. Our findings suggested that the maintenance of Golgi ribbon structure requires normal retrograde trafficking from the early endosome to the TGN, which is likely to be mediated by the formation of VAMP4-containing SNARE complexes.

## Introduction

The mammalian Golgi apparatus is a highly complex, multi-compartment organelle. At the ultrastructural level, the Golgi apparatus comprises flattened membrane-bound compartments termed cisternae, which are apposed to one another to form a Golgi stack. Within the polarized structure of a Golgi stack, proteins and lipids are exchanged with the endoplasmic reticulum (ER) at the *cis* face, whereas the *trans* face communicates with the plasma membrane and compartments of the endocytic pathway [1–3].

In vertebrate cells, a typical Golgi apparatus appears as a twisted ribbon-like network, termed the *Golgi ribbon,* which is located in the juxtanuclear and pericentriolar regions of the cell. This structure undergoes continuous lateral fission and fusion and therefore has a metastable architecture. Various factors maintain the metastable state of the Golgi ribbon, including the cytoskeleton, Golgi structural proteins, and membrane-trafficking molecules [2, 4–6]. Inhibition of these molecules breaks down the pericentrosomal Golgi ribbon into discrete fragments and highly dispersed elements, a process termed fragmentation. At the onset of mitosis, the Golgi apparatus undergoes fragmentation, and the resulting vesicles are partitioned equally between the two daughter cells. Following cytokinesis, these vesicles subsequently fuse to form a new Golgi apparatus [7]. Although the Golgi apparatus has been intensively studied for decades, the molecular mechanism(s) by which this organelle maintains its characteristic structure is still not fully understood.

Trafficking between different intracellular membrane compartments involves transport-vesicle intermediates, which are generated at the donor membranes and then delivered to specific acceptor membranes [8–10]. Targeting and fusion between various intracellular membrane vesicles are precisely controlled by many proteins, including coat proteins, Rab small GTPases, Rab effectors, and SNAREs (soluble *N*-ethylmaleimide-sensitive fusion factor attachment protein receptors) [8, 11, 12]. SNARE proteins, which are highly conserved among eukaryotes, are involved in docking and fusion processes during intracellular vesicle trafficking [8, 13, 14]. Members of the SNARE family are characterized by the presence of a common structural domain, the SNARE motif [15, 16]. SNAREs are structurally and functionally designated as either vesicle (v)-SNAREs or target-membrane (t)-SNAREs. For a given transport event, it is generally believed that each family contributes one or two SNARE motifs, such that four SNARE motifs form a four-helix–bundle structure referred to as the *trans*-SNARE complex [17–19]. Within the cell, different combinations of SNAREs generate a wide spectrum of *trans*-SNARE complexes, each of which then mediates specific transport events [20, 21].

Various types of SNARE proteins are localized in the Golgi apparatus. One member of the family, vesicle-associated membrane protein 4 (VAMP4), is predominantly localized in the *trans*-Golgi network (TGN), although it is also found in endosomes [22–24]. Furthermore, VAMP4 is the only Golgi-localized v-SNARE that exhibits restricted expression in vertebrates [13, 25]. Using co-immunoprecipitation analysis, Mallard and colleagues [23] identified the putative t-SNAREs syntaxin 6, syntaxin 16, and Vti1a as necessary cognates of VAMP4. These SNAREs are part of the SNARE complexes that mediate the retrograde trafficking step of the Shiga toxin B-subunit (STxB) and TGN38/46 from the early endosome (EE) to the TGN [23, 26]. In PC12 and AtT-20 cells, VAMP4 participates in the maturation of secretory vesicles from the TGN [27, 28].

In this study, we found that VAMP4 depletion resulted in Golgi fragmentation. The resulting Golgi fragments remained in the juxtanuclear area and maintained normal stacking. In addition, anterograde transport was not impaired in VAMP4-depleted cells, which contained intact microtubule arrays. Furthermore, depletion of not only VAMP4 but also the cognate SNARE partners of VAMP4 induced Golgi fragmentation. These results suggest that the maintenance of the Golgi ribbon structure requires normal retrograde trafficking from the EE to the TGN, which is likely to be mediated by the formation of VAMP4-containing SNARE complexes.

## Materials and methods

### Antibodies

The following antibodies were used in the study: mouse monoclonal antibodies against GM130, Vti1a, GS15 (BD Biosciences, Bedford, MA, USA), β-actin, β-tubulin, acetylated α-tubulin (Sigma, St Louis, MO, USA), Golgin-97 (Invitrogen, Carlsbad, CA, USA), and a lumenal epitope of VSV-G (clone 8G5; kindly provided by D. Lyles, Wake Forest University School of Medicine, NC, USA); rabbit monoclonal antibodies against GM130 (Abcam, Cambridge, United Kingdom); rabbit polyclonal serum against VAMP4 (Synaptic Systems, Göttingen, Germany); and rabbit polyclonal antibodies against syntaxin 6 and syntaxin 16 (Synaptic Systems). The following secondary antibodies were used: Cy3-conjugated goat anti-mouse IgG and Cy3-conjugated goat anti-rabbit IgG (Jackson ImmunoResearch Laboratories, West Grove, PA, USA); Alexa Fluor 488-conjugated goat anti-mouse IgG, Alexa Fluor 488-conjugated goat anti-rabbit IgG, and Alexa Fluor 405–conjugated goat anti-rabbit IgG (Molecular Probes, Eugene, OR, USA).

### Cell culture, transfections, and small interfering RNAs (siRNAs)

HeLa cells and HeLa cells expressing the Golgi-stack enzyme *N*-acetylgalactosaminyltransferase-2 (GalNAc-T2) fused to the green fluorescent protein (GFP) (GalNAc-T2-GFP) (a kind gift from B. Storrie, University of Arkansas for Medical Sciences, AR, USA, and H. Hashimoto, Fukushima Medical University School of Medicine, Fukushima, Japan) were grown in recording chambers consisting of 7 × 7 mm plastic cylinders containing fibronectin-coated coverslips (BD Biosciences). Cells were cultured in Dulbecco’s modified Eagle’s medium (DMEM, Sigma) supplemented with 10 % bovine calf serum (HyClone, Logan, UT, USA), 100 U/ml penicillin-G, and 100 μg/ml streptomycin (Gibco BRL, Gaithersburg, MD, USA) at 37 °C under 5 % CO_2_. For gene silencing, 100–200 nM siRNA against each SNARE protein, or the same concentration of a control siRNA, was introduced into the HeLa cells using the DMRIE-C Transfection Reagent (Invitrogen). Three days after siRNA transfection, cells were either processed for immunofluorescence microscopy or lysed for Western blotting. To deplete syntaxin 6 efficiently, two consecutive transfections were performed, with the second transfection 48 h after the first. The cells were analyzed 48 h after the second transfection. The following siRNA oligonucleotides, used to target VAMP4, syntaxin6, and Vti1a, were provided by Ambion (Austin, TX, USA): VAMP4 siRNA-1, 5′-AGCTTATCGGATAATGCAA-3′ (designed by Ambion); VAMP4 siRNA-2, 5′-GGGACCATCTGGACCAAGA-3′ [29]; syntaxin6 siRNA, 5′-GCAACTGAATTGAGTATAA-3′ (designed by Ambion); Vti1a siRNA, 5′-GGGATGTACAGCAACAGAA-3′ (designed by Ambion); syntaxin 16, 5′-GCAGCGATTGGTGTGACAA-3′ [26]; and Control siRNA (a non-specific sequence designed by Ambion, used as a negative control).

### Western blotting

Sample preparation for Western blotting was performed as described previously [30]. The blots were incubated first with primary antibodies and then with secondary IgG antibodies conjugated with horseradish peroxidase (HRP). HRP activity of the secondary antibodies was visualized with the Immobilon™ Western Chemiluminescent HRP Substrate (Merck KGaA, Darmstadt, Germany). Images were captured and analyzed using an ATTO Cool Saver (ATTO, Tokyo, Japan).

### Immunofluorescence cell staining

HeLa cells grown in recording chambers were fixed in phosphate-buffered saline (PBS) containing 4 % paraformaldehyde (PFA) for 5 min, permeabilized with 0.1 % Triton X-100 in PBS, and blocked overnight with Block Ace (Snow Brand Milk Products Co., Ltd., Tokyo, Japan). Cells were then incubated with the appropriate primary antibody for 1 h. After being washed several times, the cells were labeled with the appropriate secondary antibodies for 1 h. In some cases, cells were stained with Hoechst-33342 (Dojin Laboratories, Kumamoto, Japan) to visualize the nuclei.

### Fluorescence recovery after photobleaching (FRAP)

To mark the Golgi in HeLa cells, the HeLa cells expressing GalNAc-T2-GFP were transfected with control or VAMP4 siRNAs. Three days later, the cells were used for FRAP experiments, performed using a confocal microscope at room temperature. A region of interest was bleached with 100 % laser power for three frames, and the fluorescence recovery was monitored for 75 frames every 2 s. Quantitative analysis of these images was performed using the LaserSharp 2000 software (Carl Zeiss, Oberkochen, Germany). The fluorescence intensity in the bleached region was normalized against that of an unbleached region in the vicinity, and the results were further normalized against the pre-bleached intensity.

### Vesicular stomatitis virus glycoprotein (VSV-G)–GFP transport assay

HeLa cells were transfected with control or VAMP4 siRNAs. Two days later, the cells were transfected with GFP-tagged VSV-G derived from a temperature-sensitive mutant strain (ts045) of vesicular stomatitis virus (ts045 VSV-G-GFP; a kind gift from K. Simons, Max Planck Institute, Dresden, Germany) and incubated at 40 °C overnight to accumulate VSV-G in the ER. On the following day, the cells were shifted to 32 °C for 0, 20, 40, or 60 min to allow exit of VSV-G from the ER and then stained with a mouse monoclonal anti-VSV-G antibody against a lumenal epitope of VSV-G (clone 8G5). The cells were washed with cold PBS and fixed with 4 % PFA at room temperature. A Cy3-conjugated goat anti-rabbit IgG was used as the secondary antibody. To assess the reduction in VAMP4 protein, the cells were stained with polyclonal anti-VAMP4 antibodies, and then visualized with an Alexa Fluor 405–conjugated anti-rabbit secondary antibody. The ratio of surface-to-total VSV-G–GFP fluorescence was calculated using the ImageJ 1.42 software (http://rsbweb.nih.gov/ij).

### Confocal microscopy

Fluorescent images were acquired using the Digital Eclipse C1si-Ready confocal microscope system (Nikon, Tokyo, Japan), which comprised an inverted microscope (Nikon TE2000-E) equipped with a Nikon Plan Fluor 40×/1.3 oil-immersion objective lens. A solid-state laser (408 and 488 nm) and a He–Ne laser (543 nm) were used for three-color excitation. Alexa Fluor 405 and Hoechst-33342 fluorescence were passed through the 450/35-nm band-pass filter, GFP and Alexa Fluor 488 fluorescence were passed through the 515/30-nm band-pass filter, and Cy3 fluorescence was passed through the 605/75-nm band-pass filter. The emitted fluorescence was acquired with a back-illuminated EM-CCD camera (iXON DU-897; Andor Technology, Belfast, UK) driven by the EZ-C1 software (Nikon). *Z* steps (1 μm) were driven by a built-in *z*-axis motor (Nikon). For FRAP analysis, GalNAcT2-GFP fluorescence was visualized with a 488-nm excitation beam, and fluorescence images were acquired with a Radiance 2100 (Carl Zeiss, Oberkochen, Germany) confocal microscope system attached to an inverted microscope (TE2000, Nikon, Tokyo, Japan), which was equipped with a Nikon Plan Apo 60 × H/1.40 oil-immersion objective lens.

### Image analysis

To quantify the degree of Golgi fragmentation, control, VAMP4, syntaxin 6, syntaxin 16, and Vti1a siRNA-treated cells were double-stained with antibodies against GM130 and each SNARE protein. The depletion of SNAREs was confirmed by staining for each protein, and the Golgi structures were visualized by staining for GM130. The number of Golgi objects per cell was determined after applying a fixed threshold using the “MultiThresholder” and “Analyze Particle” plugins in ImageJ 1.42.

### Transmission electron microscopy

HeLa cells were fixed overnight using a mixture of 2 % PFA and 2 % glutaraldehyde in 10 mM PBS, pH 7.4. After washing with PBS, the cells were post-fixed in 1 % osmium tetroxide diluted with PBS. The samples were dehydrated through an increasing ethanol series and embedded in Epon 812 resin (TAAB, Berkshire, England). Ultrathin sections were cut and double-stained with uranyl acetate and lead citrate and then observed under a transmission electron microscope (JEM-1010, JEOL, Tokyo, Japan).

### Statistical analysis

Data represent the mean ± SE of all cells examined in at least three independent experiments. Statistical significance was assessed using Tukey’s post hoc test or the Kruskal–Wallis test. A *p* value <0.01 was considered statistically significant.

## Results

### Depletion of VAMP4 causes fragmentation of the Golgi apparatus

**Fig. 1:**
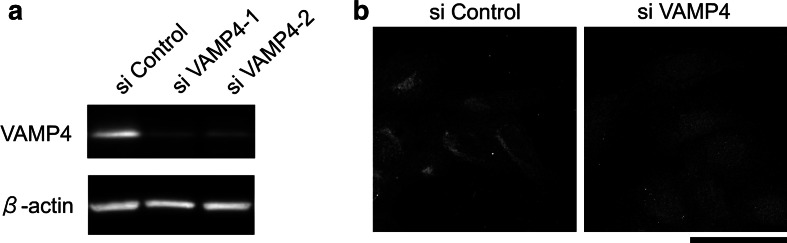
Knockdown of VAMP4 using RNAi. **a** HeLa cells were treated with a control siRNA or two separate siRNAs against VAMP4. Total cell extracts were analyzed by Western blotting with an anti-VAMP4 antibody. β-Actin was used as a loading control. **b** Knockdown of VAMP4 by VAMP4 siRNA-1 was assessed by indirect immunofluorescence using an anti-VAMP4 antibody. Immunoreactivity of the VAMP4 protein is absent in siRNA-knockdown cells. *Bar* 50 μm

To define the role of VAMP4 in maintaining the structure of the Golgi apparatus, we depleted VAMP4 by RNA interference (RNAi). Two different target sequences were synthesized, and the efficiency of VAMP4 depletion was examined by Western blotting and immunocytochemical analyses. Preliminary experiments demonstrated that the protein level of VAMP4 exhibited little reduction for 24 h following siRNA transfection, after which it declined steadily over the next 48 h, reaching a minimum 72 h after transfection. Treatment with either siRNA significantly reduced the levels of VAMP4 protein relative to the level in cells treated with a random siRNA control sequence (Fig. 1a): quantification of the degree of siRNA-mediated depletion indicated that the VAMP4 protein level was reduced to 6–32 % of the control level (data not shown). Immunocytochemical analysis confirmed that the expression of VAMP4 was downregulated in RNAi-induced cells (Fig. 1b).

**Fig. 2:**
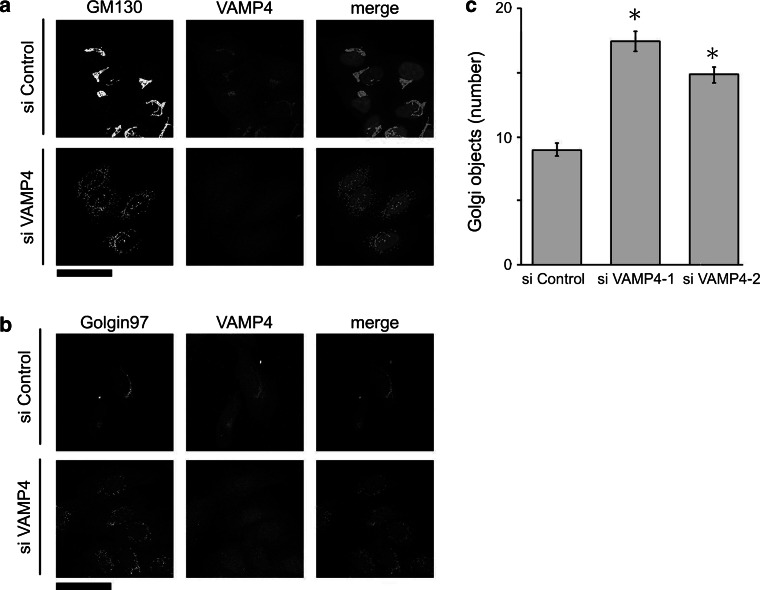
Depletion of VAMP4 causes the Golgi apparatus to fragment. **a** HeLa cells were treated with a control siRNA (*top*) or VAMP4 siRNA-1 (*bottom*) and then double-stained with a rabbit monoclonal anti-GM130 antibody (*left, green*) and an anti-VAMP4 antibody (*middle, red*). The nuclei were counterstained with Hoechst-33342 (*blue*). Images shown in the *z*-plane are all maximum-intensity projections of confocal image stacks through the entire cell. *Bar*, 50 μm. **b** HeLa cells were treated with a control siRNA (*top*) or VAMP4 siRNA-1 (*bottom*) and then double-stained with an anti-Golgin-97 antibody (*left, green*) and an anti-VAMP4 antibody (*middle, red*). Images shown in the *z*-plane are all maximum-intensity projections of confocal image stacks through the entire cell. *Bar*, 50 μm. **c** The number of distinct Golgi objects per cell was estimated using fixed acquisition parameters and image thresholding, followed by counting using the “Analyze Particles” function in ImageJ. Values represent the mean ± SE from three independent experiments (*n* = 300 cells in each case) (*p < 0.01). (Color figure online)

To examine the effect of VAMP4 depletion on the architecture of the Golgi apparatus, we used immunocytochemical methods to investigate the intracellular localization of two Golgi protein markers, GM130 (a *cis*/medial-Golgi marker) and Golgin-97 (a *trans*-Golgi marker). The distributions of these proteins revealed that the normal distribution of the Golgi ribbon structure was juxtanuclear, with a relatively compact distribution that wrapped around the nucleus in some cells (Fig. 2a, b). By contrast, the Golgi apparatus of cells treated with VAMP4 siRNA was organized into discrete small elements (*Golgi fragments*) that were not uniformly distributed throughout the cytoplasm, but remained in the juxtanuclear area (Fig. 2a, b). To define the nature of these Golgi fragments more precisely, we quantified the number of Golgi fragments per cell. Compared with control cells, VAMP4-depleted cells contained a significantly higher average number of distinct fluorescent Golgi objects (Fig. 2c). Because the two independent siRNA target sequences yielded the same phenotype (Fig. 2c), off-target effects resulting from the RNAi treatment itself were unlikely. Next, we performed immunocytochemical analysis of other Golgi-resident proteins, including the medial-Golgi t-SNARE GS15; the *trans*-Golgi t-SNAREs syntaxin 6, syntaxin 16, and Vti1a; and the galactosyltransferase GalNac-T2. In VAMP4-depleted cells, these Golgi-resident proteins were present on fragmented Golgi membranes (data not shown).

**Fig. 3:**
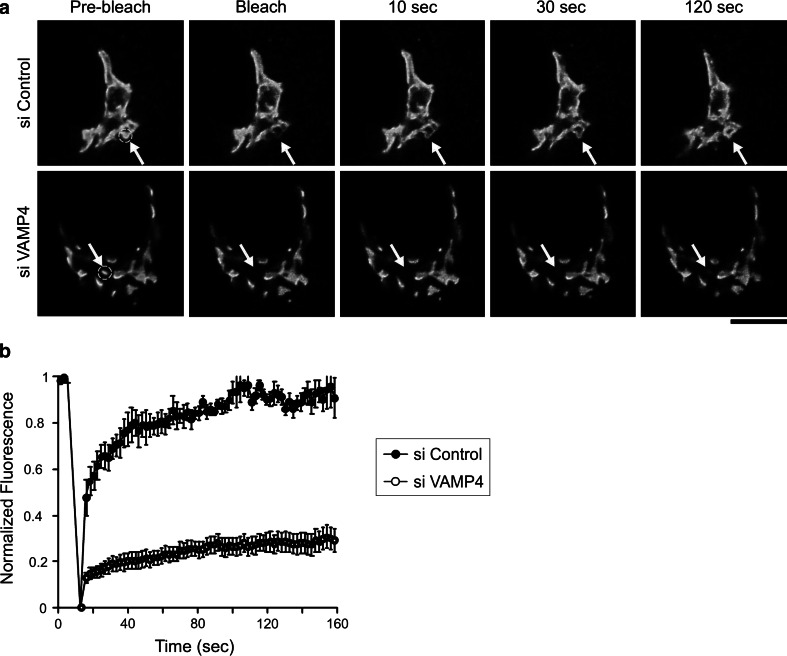
VAMP4 depletion disrupts the connections between Golgi stacks. **a** HeLa cells expressing GalNAcT2-GFP were treated with a control siRNA (*top*) or VAMP4 siRNA-1 (*bottom*). After 72 h, the area of the Golgi indicated by the *arrow* was bleached. Fluorescence recovery was followed for 160 s. Representative images at the indicated *times* are shown. **b** Fluorescence recovery was quantified by dividing GFP fluorescence within the bleached spot by the fluorescence of a nearby unbleached region in the same Golgi object. Each curve was normalized by the values between a minimum of 0 and a maximum of 1. The rates of recovery in control siRNA-treated cells (*black circles*) and VAMP4 siRNA-treated cells (*white circles*) are plotted. The results show the mean fluorescence recovery ± SE from five to six cells from three independent experiments. *Bar*, 10 μm

To determine whether the Golgi fragments were connected to each other, we performed FRAP experiments in HeLa cells stably expressing the Golgi enzyme *N*-acetyl galactosaminyltransferase-2 tagged with green fluorescent protein (GalNAcT2-GFP). A defined region of the Golgi was bleached, and fluorescence recovery was recorded for 160 s. The GalNAcT2-GFP fluorescence recovered rapidly after photobleaching in control cells, but not in VAMP4-depleted cells (Fig. 3a, b). Based on these observations, we concluded that there was little interconnection between the Golgi elements in the VAMP4-depleted cells.

### Golgi stacking and anterograde transport do not require VAMP4

**Fig. 4:**
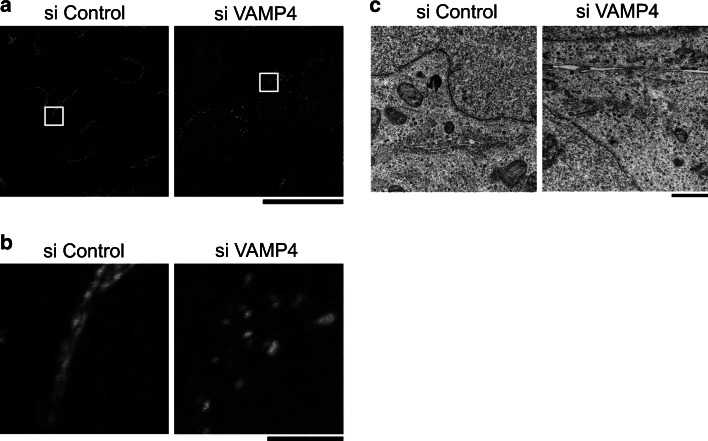
Depletion of VAMP4 converts the Golgi into ministacks. **a** HeLa cells were treated with a control siRNA (*top*) or VAMP4 siRNA-1 (*bottom*) and then double-stained with a rabbit monoclonal anti-GM130 antibody (*green*) and an anti-Golgin-97 antibody (*red*). Images shown in the *z*-plane are all maximum-intensity projections of confocal image stacks through the entire cell. *Bar*, 50 μm. **b** An enlarged selection from each of the images above (*white squares*). *Bar*, 5 μm. **c** Transmission electron micrographs of the juxtanuclear region in control siRNA (*right*) or VAMP4 siRNA-1 (*left*)-treated HeLa cells. *Bar*, 1 μm. (Color figure online)

To characterize in detail the Golgi phenotype of VAMP4-depleted cells, we closely examined cells double-stained with antibodies against GM130 (a *cis*/medial-Golgi marker) and Golgin-97 (a *trans*-Golgi marker). The two proteins maintained a distinct distribution both in the Golgi ribbon structure of control cells and in the Golgi fragments of VAMP4-depleted cells (Fig. 4a, b). To confirm that these stacks were compartmentalized, we analyzed the cells by transmission electron microscopy. In the absence of VAMP4, although the length of the Golgi stack was shortened, the stacking appeared normal (Fig. 4c). These results suggest that VAMP4 plays an important role in maintaining the Golgi ribbon structure, but appears to have no function in cisternal stacking.

**Fig. 5:**
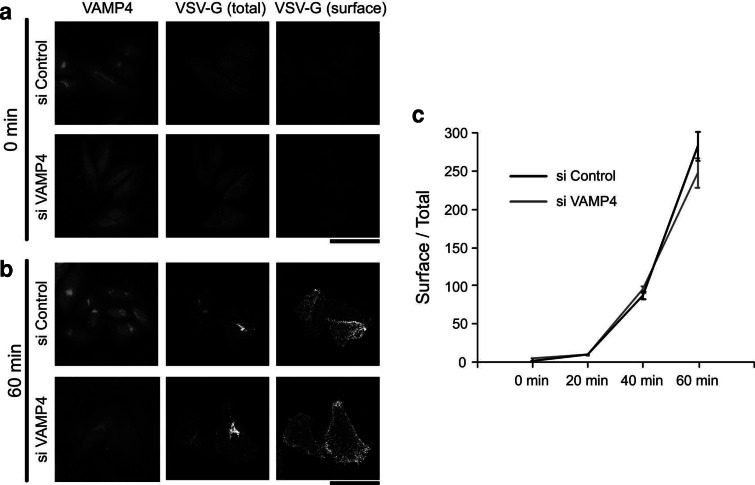
Anterograde transport in VAMP4-depleted cells. **a**, **b** HeLa cells were transfected with control (*top*) or VAMP4-1 (*bottom*) siRNAs. Two days after siRNA transfection, the cells were transfected with ts045 VSV-G-GFP and incubated at 40 °C overnight. Cells were shifted to 32 °C for 0 min (**a**) or 60 min (**b**), transferred to ice, and exposed to anti-VSV-G before fixation. Total VSV-G was measured using GFP fluorescence (*middle*), whereas surface VSV-G was measured by staining live cells with a monoclonal antibody against the lumenal domain of VSV-G (*right*). Depletion of VAMP4 was assessed by staining with an anti-VAMP4 antibody (*left*). *Bar*, 50 μm. **c** Surface and total fluorescence values of the *z*-stacked images were quantified and expressed as ratios to indicate the relative amount of VSV-G on the plasma membrane. Data represent the mean ± SE from three to four independent experiments (*n* = 50–100 cells in each case)

Next, we tested the effects of VAMP4 depletion on anterograde transport. To this end, we monitored the trafficking of a variant of VSV-G derived from the temperature-sensitive ts045 mutant strain of vesicular stomatitis virus. VSV-G is a well-characterized marker for protein trafficking that accumulates in the ER at 40 °C, but is transported to the plasma membrane after cells are shifted to the permissive temperature (32 °C) [31]. In both control and VAMP4-depleted cells, VSV-G accumulated in the ER at 40 °C (Fig. 5a); after the shift to 32 °C, it was present at the cell surface within 60 min (Fig. 5b). Quantitative analysis of the ratio between surface and total VSV-G fluorescence indicated that VSV-G trafficking was normal in the absence of VAMP4 (Fig. 5c). Normal trafficking was confirmed further using HeLa cells expressing a secreted luciferase from *Cypridina noctiluca* (CLuc). As expected, CLuc secretion was normal in VAMP4-depleted cells (data not shown). These results indicate that VAMP4 depletion did not inhibit anterograde transport.

### VAMP4-depleted cells contain intact microtubule arrays

**Fig. 6:**
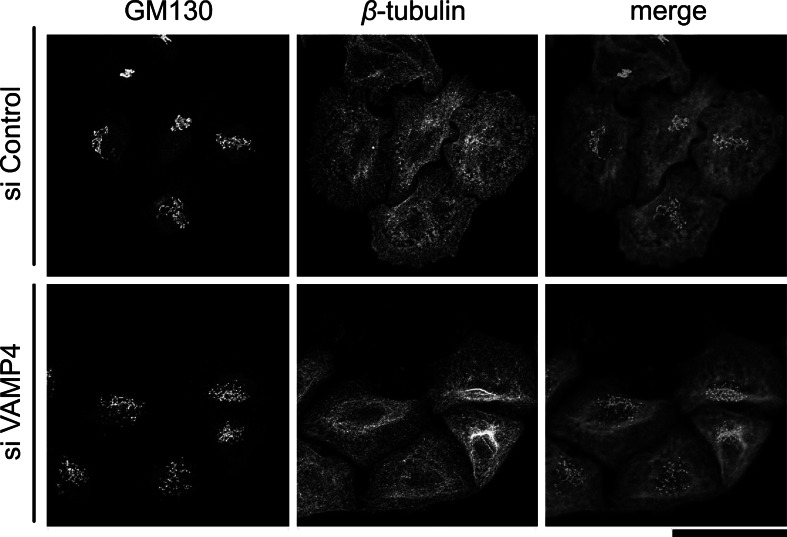
Microtubule integrity is maintained in VAMP4-depleted cells. Microtubules were examined in HeLa cells 72 h after treatment with the control siRNA (*top*) or VAMP4 siRNA-1 (*bottom*) and then double-stained with antibodies against GM130 (*left, green*) and *β*-tubulin (*middle, red*). Images shown are all maximum-intensity projections of confocal image stacks in the *z*-plane through the entire cell. *Bar*, 50 μm. (Color figure online)

In HeLa cells, Golgi stacks are normally juxtaposed with the centrosome, the major organizing center for microtubules in the cytoplasm [32], and microtubule depolymerization leads to the redistribution of the Golgi apparatus from the centrosome to peripheral ER protein exit sites [33]. Therefore, we next asked whether the Golgi fragmentation induced by VAMP4 depletion was associated with changes in the arrangement of cytoplasmic microtubules; however, staining for β-tubulin revealed no microtubule depolymerization in VAMP4-depleted cells. Instead, most VAMP4-depleted cells exhibited the well-defined microtubule-organizing centers typical of normal interphase cells (Fig. 6). In addition, staining for stable, acetylated α-tubulin revealed no obvious reduction in stable microtubules in VAMP4-depleted cells (data not shown).

### Depletion of the cognate SNARE partners of VAMP4 causes fragmentation of the Golgi apparatus

**Fig. 7:**
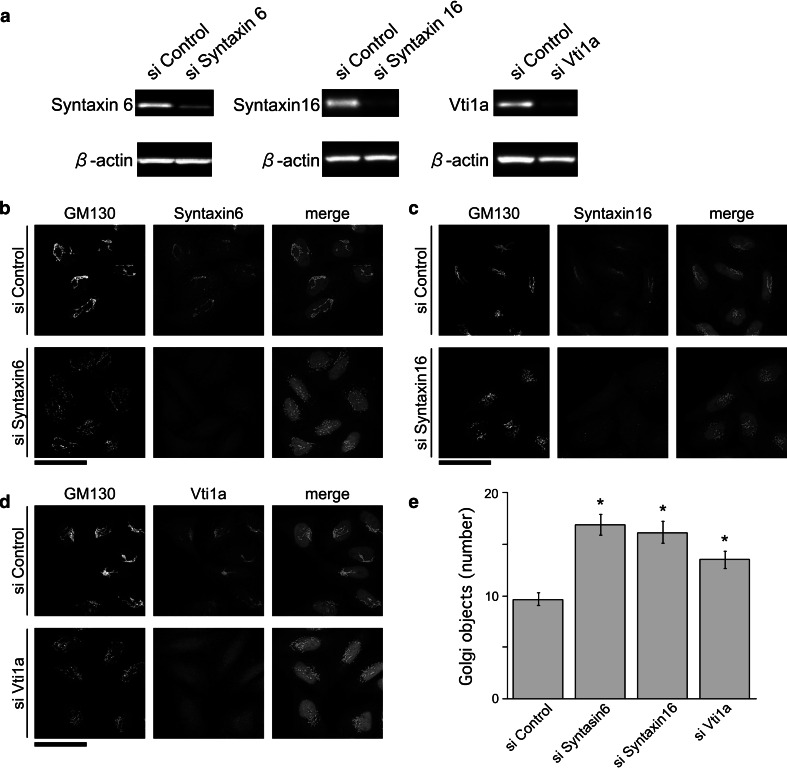
Golgi fragmentation by depletion of the VAMP4-cognate binding partners. HeLa cells were treated with control, syntaxin 6, syntaxin 16, or Vti1a siRNAs. **a** Total cell extracts were analyzed by immunoblotting using antibodies against syntaxin 6 (*left*), syntaxin 16 (*middle*), or Vti1a (*right*). β-actin was used as a loading control. **b**, **c**, **d** Golgi structures in siRNA-treated cells were analyzed by immunostaining using antibodies against GM130 (**b**–**d**, *left*), syntaxin 6 (**b**, *middle*), syntaxin 16 (**c**, *middle*), or Vti1a (**d**, *middle*). Images shown in the *z*-plane are all maximum-intensity projections of confocal image stacks through the entire cell. *Bar*, 50 μm. **e** The number of distinct Golgi objects per cell was estimated using fixed acquisition parameters and image thresholding, followed by counting using the “Analyze Particles” function in ImageJ. Values represent the mean ± SE from three independent experiments, *n* = 100–200 cells in each case. (*p < 0.01)

VAMP4 interacts with syntaxin 6, syntaxin 16, and Vti1a to form a SNARE complex that mediates trafficking of the post-Golgi vesicles from the EE back to the TGN [23]. Therefore, we investigated the roles of these t-SNAREs in the maintenance of the Golgi ribbon structure. Treatment of HeLa cells for 72–96 h with siRNAs against syntaxin 6, syntaxin 16, or Vti1a significantly depleted the corresponding proteins, as revealed by immunoblotting (Fig. 7a). The Golgi phenotype of these t-SNARE–depleted cells closely resembled that of after VAMP4-depleted cells (Fig. 7b–d), and quantitative analysis revealed that the degree of Golgi fragmentation was also similar (Fig. 7e). These results suggested that VAMP4-containing SNARE complexes are required for maintenance of the Golgi ribbon structure.

## Discussion

The architecture of the mammalian Golgi apparatus is called the Golgi ribbon. This structure is only observed in vertebrates. By contrast, in many simpler eukaryotes, the Golgi apparatus is dispersed throughout the cytoplasm [2, 5, 34]. During mitosis, the mammalian Golgi ribbon structure is remodeled into fragments, and it reassembles into a ribbon structure following cytokinesis [7]. The mammalian Golgi contains various molecules that link and maintain its ribbon structure, e.g., membrane-trafficking molecules, cytoskeletal proteins, and Golgi structural proteins, and the loss or inhibition of these molecules induces Golgi fragmentation [2, 5]. However, most of these molecules are present not only in cells that can form a Golgi ribbon but also in plant, yeast, and insect cells, which lack the ribbon structure [35]. The membrane-trafficking molecule VAMP4 is an exception as it is only present in vertebrates [13, 25]. Therefore, we depleted VAMP4 to examine this protein’s role in the maintenance of the Golgi ribbon structure.

Immunocytochemical analyses revealed that in the absence of VAMP4, the continuity of the Golgi ribbon was lost, but the Golgi fragments remained localized in the perinuclear area (Fig. 2). This phenotype is termed *central Golgi fragmentation*, and it is also observed following depletion of the Golgi-localized tethering factors GM130, Cog3p, and GCC185 [6, 36–38]. In addition, depletion of VAMP4’s cognate SNARE partners (syntaxin 6, syntaxin 16, and Vti1a) caused a similar phenotype (Fig. 7). Furthermore, under physiological conditions, central Golgi fragmentation is observed in late G2 phase when Golgi stacks lose their interconnectivity [39]. By contrast, when the microtubules are depolymerized by nocodazole or when the function of p115 is inhibited, Golgi fragments become dispersed throughout the cytoplasm [32, 33, 40, 41], a phenomenon referred to as peripheral Golgi fragmentation [2]. The perinuclear positioning of the Golgi ribbon is imposed by the cytoskeleton and the activities of motor proteins [2] and it is likely that depletion of VAMP4 does not affect their functions. This idea is supported by our observation that VAMP4-depleted cells contained intact microtubule arrays (Fig. 6).

In both G2-phase and VAMP4-depleted cells, the Golgi structures exhibited fragmentation. Therefore, we examined the possibility that VAMP4 depletion would induce the Golgi fragmentation in G2 phase. Data compiled in Cyclebase.org, a comprehensive multi-organism online database of cell-cycle experiments [42], indicate that the expression levels of VAMP4 do not change during the cell cycle. Thus, the data available so far indicate that VAMP4 is not involved in Golgi fragmentation during mitosis.

Detailed analyses of the fragmented Golgi revealed that in the absence of VAMP4, the length of the Golgi stack was shortened, but Golgi stacking and compartmentalization were normal (Fig. 4). Similar Golgi “ministacks” are also observed when the microtubules are depolymerized following nocodazole treatment or in the absence of the Golgi-localized tethering factors Cog3p and GCC185 [6, 36, 43]. By contrast, depletion of the Golgi structural proteins GRASP55 and GRASP65 (Golgi reassembly stacking proteins of 55 and 65 kD) results in not only Golgi fragmentation but also cisternal unstacking [44]. The fragmented Golgi phenotypes of VAMP4-depleted cells, including central Golgi fragmentation and formation of ministacks, resembled the phenotype resulting from silencing of the tethering factors Cog3p and GCC185. VAMP4, Cog3p, and GCC185 regulate the same trafficking pathway, which involves retrograde transport between the TGN and the EE; based on the results described above, they may participate in common mechanisms of Golgi fragmentation.

The Golgi apparatus is the central organelle involved in membrane trafficking, and continuous vesicular transport reactions are observed among the ER, ER-Golgi intermediate complex (ERGIC), and *cis*-Golgi; between the *trans*-Golgi and endosome/plasma membrane; and within the Golgi. An alteration in the normal balance between anterograde and retrograde membrane pathways leads to the disruption of Golgi organization [45, 46]. For example, treatment with brefeldin A, an inhibitor of Golgi/TGN-associated coated vesicle formation, inhibits ER-Golgi anterograde transport, but not the retrograde pathway. As a consequence, the Golgi complex disassembles following exposure to this inhibitor [47]. During mitosis, anterograde transport between the ER and Golgi is inhibited, whereas retrograde transport continues [48, 49], and this imbalanced membrane trafficking accelerates Golgi disassembly at the onset of mitosis. The major role of VAMP4 in membrane trafficking is the regulation of retrograde transport from the EE to the TGN. Indeed, dominant-negative or inhibitory antibodies against VAMP4 impair the retrograde transport of STxB and TGN38/46 from the plasma membrane to the TGN through the EE [23]. In this study, we showed that anterograde transport of VSV-G from the TGN to the plasma membrane is functional in VAMP4-depleted cells (Fig. 5). Therefore, VAMP4-depleted cells are likely to have an imbalance in post-Golgi vesicle transport between the TGN and plasma membrane. Furthermore, as mentioned above, depletion of Cog3p or GCC185 causes Golgi fragmentation, similar to the phenotype of VAMP4-depleted cells; cells depleted of any of these proteins also exhibit an imbalance in post-Golgi vesicle transport [6, 36]. These observations suggest that (1) a normal balance in membrane transport between the TGN and the EE/plasma membrane interface is required to maintain the Golgi ribbon structure, and (2) depletion of VAMP4 perturbs SNARE-mediated membrane fusion between EE-derived vesicles and the TGN, and may thereby induce Golgi fragmentation.
